# 18F-Prostate-Specific Membrane Antigen and 18F-Fluorodeoxyglucose PET/CT Unmasked the Characteristics of Prostate Lymphoma: A Case Report and Literature Review

**DOI:** 10.3389/fmed.2022.842093

**Published:** 2022-04-06

**Authors:** Fan Jiang, Junjie Fan, Hua Liang, XiaoYi Duan, Dalin He, Kaijie Wu

**Affiliations:** ^1^Department of Urology, First Affiliated Hospital of Xi’an Jiaotong University, Xi’an, China; ^2^Department of Urology, Baoji Central Hospital, Baoji, China; ^3^Department of Pathology, First Affiliated Hospital of Xi’an Jiaotong University, Xi’an, China; ^4^Department of Radiology, First Affiliated Hospital of Xi’an Jiaotong University, Xi’an, China

**Keywords:** prostate lymphoma, PSMA, PET/CT, clinical symptom, management

## Abstract

Prostate lymphoma (PL) is rarely observed and may be concurrently presented with prostate adenocarcinoma. Moreover, the appearance of PL on conventional imaging is similar with prostate adenocarcinoma. Thus, most of PL is diagnosed through prostate biopsy, or accidentally found in the specimens of surgery. Prostate-specific membrane antigen (PSMA) PET/CT has improved the management of prostate adenocarcinoma. While, the question regarding whether it benefits the discovery of the characteristics of PL is unknown. A 32-year-old man presented with worsening dysuria for 1 month, and the prostate-specific antigen (PSA) concentration was normal. While the pelvic MRI showed a mass in the prostate and multiple enlarged lymph nodes in the bilateral inguinal area. Then, the diagnosis of prostate adenocarcinoma was considered, but the serum PSA was normal and he was younger than most patients. So, 18F-PSMA PET/CT was then performed to further reveal the characteristics of the lesion and guide biopsy. However, there was no abnormal PSMA uptake in the lesion of the prostate and lymph nodes of the pelvic cavity and bilateral inguinal area. These lesions presented with increased glucose metabolism on fluorodeoxyglucose (FDG) PET/CT, and the prostate biopsy was then performed. PL was confirmed based on the results of the histopathologic examination, and the patient subsequently received systemic chemotherapy plus radiotherapy. Fortunately, the symptoms and the lesions completely disappeared after radiotherapy. The clinical symptoms of PL are atypical, and PL and adenocarcinoma may be concurrently presented. Moreover, distinguishing PL from prostate adenocarcinoma based on the appearance of conventional imaging is difficult. As opposed to prostate adenocarcinoma, a high FDG-avidity and low PSMA uptake by lymphoma either in the prostate or metastases are seen. So, PSMA PET/CT combined with FDG PET/CT can non-invasively identify the characteristics and origin of PL.

## Introduction

Prostate lymphoma (PL) is rarely observed, accounting for 0.09% of all prostate neoplasms and 0.1% of all non-Hodgkin lymphomas (1). Because its clinical symptoms usually manifest as lower urinary tract symptoms, most patients are initially misdiagnosed as prostatitis or benign prostate hyperplasia and miss the optimal treatment time. Moreover, PL and adenocarcinoma may be concurrently present, and the appearance of PL on conventional imaging (i.e., MRI and CT) is similar with prostate adenocarcinoma (2, 3). Thus, most PL is diagnosed though prostate biopsy, or accidentally found in the specimens of transurethral resection of prostate or radical prostatectomy.

Prostate-specific membrane antigen (PSMA) is a type II transmembrane protein that is overexpressed on the cell membrane of nearly all prostatic cancer cells (4). Thus, PSMA is a promising and specific target for prostate adenocarcinoma imaging. In the past years, PSMA PET/CT has extremely improved the management of prostate adenocarcinoma when compared with conventional imaging, especially in the aspect of detecting metastatic spread and micrometastases (5, 6). Furthermore, as a molecular imaging examination, the expression level of PSMA on PET/CT is positively correlated with tumor stage and grade (7), which could help urologists identify patients who may need more intensive treatment and tailor treatment regimes. However, the appearance of PL on PSMA PET/CT and whether PSMA PET/CT benefits the discovery of the characteristics of PL are still unknown. Herein, we reported a case of a young man with PL who received both 18F-PSMA and 18F-fluorodeoxyglucose (FDG) PET/CT before diagnosis.

## Case Description

**FIGURE 1:**
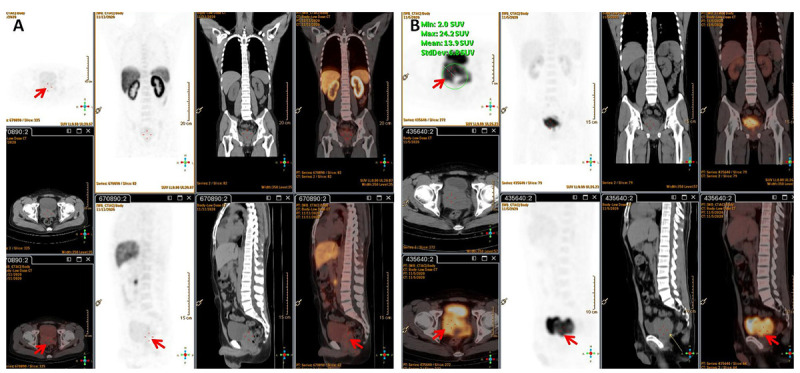
**(A)** 18F-PSMA PET/CT showed no trace PSMA uptake in the lesion (red arrow). **(B)** 18F-FDG PET/CT revealed foci with increased glucose metabolism in the prostate and bilateral seminal vesicles. Multiple enlarged lymph nodes with varying degrees of increased glucose metabolism were observed in the hilum, retroperitoneum, and pelvic cavity.

**FIGURE 2:**
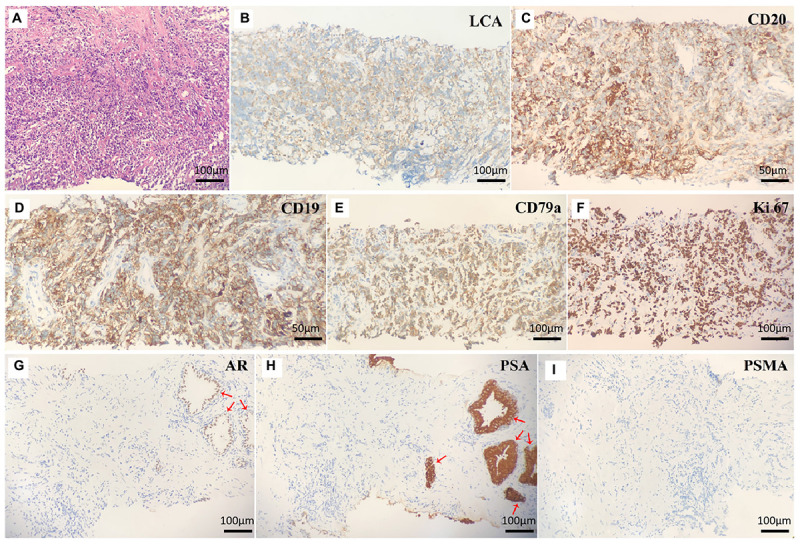
**(A)** Hematoxylin and eosin staining of prostate lesions showed diffuse large B-cell lymphoma. The eight micrographs illustrate **(B)** LCA^+^, **(C)** CD20^+^, **(D)** CD19^+^, **(E)** CD79a^+^, **(F)** Ki-67^+^, **(G)** AR^–^, **(H)** PSA^–^, and **(I)** PSMA^–^ tumor cells. Normal prostate cells were positive for AR **(G)** and PSA **(H)** (red arrow).

A 32-year-old man presented to our clinic with a 1-month history of worsening dysuria, without any fevers, night sweats, or weight loss. His past medical history included a 10-year history of smoking. His family history was unremarkable. The serum blood count was normal and the components of blood cell counts were as follows: white blood cell (WBC) count: 5.76 × 10^9^ cells/L (normal range: 3.5–9.5 × 10^9^ cells/L); red blood cell (RBC) count: 4.60 × 10^12^ cells/L (normal range: 3.8–5.1 × 10^12^ cells/L); platelet (PLT) count: 295 × 10^9^ cells/L (normal range: 125–350 × 10^9^ cells/L); neutrophil% (NEUT%): 82.4% (normal range: 40–75%); and lymphocyte% (LYMPH%): 10.8% (normal range: 20–50%). Furthermore, the serum prostate-specific antigen (PSA) concentration was 0.7 ng/ml (normal range <4 ng/mL). However, a digital rectal exam (DRE) showed a firm and enlarged prostate with normal consistency without nodule or induration, while there was not palpable lymphadenopathy either in the Troisier or inguinal areas on physical examination. Subsequently, multiparametric MRI (mp-MRI) was performed and revealed a heterogeneous mass in the right anterior lobe of the prostate. Moreover, the lesion was also extensively invading the bladder and the bilateral seminal vesicles. Meanwhile, multiple enlarged lymph nodes in the pelvic cavity and bilateral inguinal area were observed. Based on the results of imaging and physical examination, the diagnosis of prostate adenocarcinoma was subsequently considered. However, the serum PSA was normal and enlarged lymph nodes were present in the bilateral inguinal area, which is not the usual lymph node metastasis of prostate adenocarcinoma. So, 18F-PSMA PET/CT was performed to further reveal the characteristics of the lesion and guide biopsy after an informed consent form was obtained from the patient. However, there was no abnormal trace uptake in the lesion of the prostate and lymph nodes of the pelvic cavity and bilateral inguinal area on 18F-PSMA PET/CT (Figure 1A). Besides PSMA, FDG is another tracer used for PET/CT imaging examination to reveal the characteristics of prostate malignancies and obtain accurate disease staging. Therefore, after consulting with the patient, FDG PET/CT was freely performed to identify the metabolism of the prostate mass and enlarged lymph nodes. 18F-FDG PET/CT showed foci with increased glucose metabolism in the prostate and bilateral seminal vesicles. Furthermore, multiple lymph nodes in the hilum, retroperitoneum, and pelvic cavity presented with increased glucose metabolism to varying degrees, while there was no increased glucose metabolism in the liver, spleen, and bone (Figure 1B). Then, a prostate biopsy was performed and showed prostate parenchyma infiltrated by atypical medium-sized to large-sized lymphoid cells arranged in diffuse sheets. These lymphoid cells exhibited a small to moderate amount of eosinophilic cytoplasm and nuclei with irregular contours, which dispersed chromatin and occasional prominent nucleoli. As shown in Figure 2, the tumor cells were positive for leukocyte common antigen (LCA), CD20, CD19, and CD79a but negative for PSA, PSMA, androgen receptor (AR), Bcl6, Cyclin D1, CD5, and MUM1. Thus, based on the above results of the examination, diffuse large B-cell lymphoma (DLBCL) of the prostate was then diagnosed (stage IV according to the TNM classification).

**FIGURE 3:**
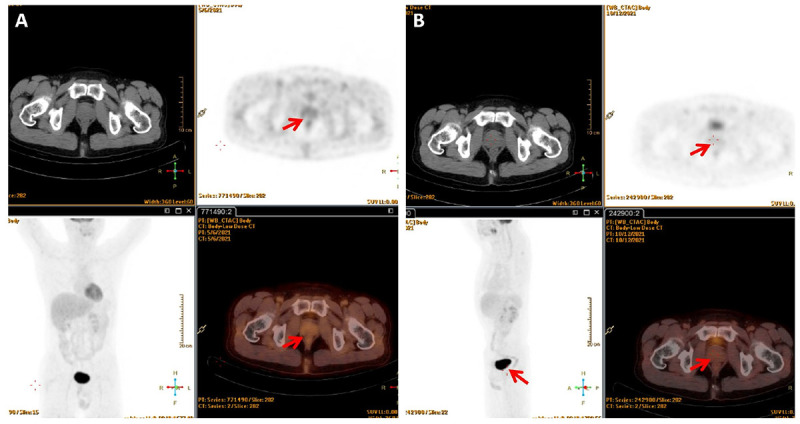
18F-FDG PET/CT showed no abnormal increased glucose metabolism in the lesion **(A)** 3 months after radiotherapy; **(B)** 6 months after radiotherapy.

Later, the patient was referred to the Department of Hematology and scheduled for bone marrow puncture in preparation for chemotherapy. The bone marrow cytology examination was normal, and fluorescence *in situ* hybridization revealed normal results for the B-cell lymphoma (BCL)-2, BCL-6, and cellular-myelocytomatosis viral oncogene (C-MYC) probes, ruling out “double-hit” or “three-hit” lymphoma. The patient chose to receive systemic chemotherapy (R-CHOP) owing to his personal preference, which consisted of a total of six cycles of three different drug regimens, including dexamethasone, cyclophosphamide, rituximab, vincristine, etoposide, and cytarabine. The patient independently completed the International Prostate Symptom Score (IPSS) patient symptom scale from before starting to after completing the treatment. Prior to chemotherapy, the patient had an IPSS score of 30. Dysuria was diminished with an IPSS score of 7 at the end of the third cycle of chemotherapy, and local radiotherapy (40 Gy, 25 cycles) was then preformed. 18F-FDG PET/CT at 3 and 6 months after radiotherapy demonstrated a complete metabolic response where the tumor mass of the prostate and enlarged lymph nodes disappeared (Figure 3). A CT scan and laboratory examination (i.e., blood routine examination, uric acid, and lactate dehydrogenase) were performed every 3 months during the follow-up period, and there was no sign of recurrence as of the 243th day after completing RT.

## Discussion

The clinical symptoms of PL are atypical. After searching PubMed-indexed biomedical journals, we identified 20 individual cases with PL reported between January 2017 and November 2021. As shown in Table 1, it usually manifests with lower urinary tract symptoms (i.e., dysuria, urine frequency, and urgency) and, in some cases, hematuria. Moreover, the serum PSA is usually within 4 ng/ml, which was consistent with previously reported (8). Thus, PL is difficult to distinguish from other prostatic diseases, such as prostatitis and benign prostate hyperplasia before biopsy. The present patient presented to our clinic with a 1-month history of worsening dysuria, and his serum PSA level was normal. Thus, he was initially suspected to have benign prostate diseases. However, a DRE showed prostate hyperplasia, disappearance of the central fissure, and a hard consistency without tenderness, which are consistent with reports of PL in the literature (9). Furthermore, mp-MRI revealed the prostate mass and the enlarged lymph nodes of the bilateral inguinal area, which is not the common metastasis site of prostate adenocarcinoma. So, 18F-PSMA and FDG PET/CT were subsequently performed to identify the characteristics of the abnormally enlarged lymph nodes and the prostate lesion, and lymphoma was then confirmed though biopsy. Therefore, even if the patient’s PSA level is relatively low, when the DRE is abnormal and enlarged lymph nodes of the pelvic cavity are present, the possibility of lymphoma should not be ignored, and PSMA PET/CT combined with FDG PET/CT before biopsy may be helpful.

**TABLE 1:** Reported cases of prostate lymphoma in the past few years.

Investigation	Years	Age	Initial symptoms	Serum PSA (ng/ml)	Diagnosed method	Origination	Imaging examination before diagnosis	Histopathology	Treatment after diagnosis	Outcome
Rallabandi et al. (19)	2021	76	LUTS and fever	Normal	TURP	Primary	MRI	IVLBCL	N.A.	N.A.
Wong et al. (16)	2021	69	Asymptomatic	2.8	Biopsy	Secondary	MRI	SLL and prostate adenocarcinoma	Ibrutinib	CR
Tlili et al. (15)	2021	66	LUTS	5.06	RP	Secondary	MRI	CLL and prostate adenocarcinoma	No	N.A.
Karademir et al. (17)	2021	60	LUTS	1	RP	Primary	Ultrasound	CLL and prostate adenocarcinoma	No	N.A.
		62	LUTS	1.87	TURP	Primary	No	MCL	R-CHOP	CR
Nerli et al. (20)	2020	73	Acute retention of urine	46.83	TURP	Primary	MRI	Follicular lymphoma	No	N.A.
Nusrat and Nazim (21)	2020	56	LUTS	1.54	Biopsy	Primary	MRI	DLBCL	R-CHOP	CR
Derigs et al. (22)	2020	28	Acute urinary retention and lower abdominal pain	Normal	Biopsy	Secondary	CT	Burkitt’s lymphoma	R-CHOP	CR
Mansbridge et al. (23)	2020	73	LUTS	2.9	TURP	Primary	CT	MBL	No	SD
El-Taji et al. (24)	2019	64	LUTS	11.06	RP	Primary	MRI	LPL and prostate adenocarcinoma	Rituximab and bendamustine	N.A.
Wang et al. (25)	2019	80	LUTS	<4	Biopsy	Primary	CT	DLBCL	Radiotherapy	SD
Galante et al. (26)	2019	77	Fever, chills, weakness, and left lower quadrant abdominal pain	5.42	TURP	Secondary	CT	BLL/CLL	Ibrutinib	PD
Jafari et al. (27)	2019	71	Hematuria and dysuria	2.1	Biopsy	Primary	CT	DLBCL	R-CHOP and radiotherapy	CR
Zhu et al. (28)	2019	71	LUTS	Normal	TURP	Primary	MRI and 18F-FDG PET/CT	IVLBCL	R-CHOP and radiotherapy	CR
Yasuoka et al. (29)	2018	73	Right lower back pain and dysuria	0.5	Biopsy	Primary	CT and MRI	DLBCL	R-CHOP	CR
Martin et al. (30)	2017	68	LUTS	1.4	TURP	Primary	Ultrasound	MZBL	R-CVP	CR
Tamang et al. (31)	2017	49	LUTS, pelvic pain, hematuria, and fever	0.4	Biopsy	Primary	CT	DLBCL	R-CHOP	CR
Milburn et al. (32)	2017	59	LUTS	1.2	TURP	Secondary	Ultrasound	MCL	No	N.A.
Hashemzadeh et al. (33)	2017	63	LUTS and hematuria	N.A.	TURP	Primary	CT	MALT	No	SD
Ezekwudo et al. (34)	2017	54	LUTS	2.03	TURP	Primary	18F-FDG PET/CT	DLBCL	R-CHOP and radiotherapy	CR

*LUTS, lower urinary tract symptoms; TURP, transurethral resection of the prostate; RP, radical prostatectomy; CR, complete response; SD, stable disease; PD, progressive disease; IVLBCL, intravascular large B-cell lymphoma; SLL, small lymphocytic lymphoma; CLL, chronic lymphocytic leukemia; MCL, mantle cell lymphoma; MBL, monoclonal B-cell lymphocytosis; LPL, lymphoplasmacytic lymphoma; MBZL, marginal zone-B lymphoma; MALT, mucosa-associated lymphoid tissue lymphoma; N.A., not available.*

Prostate lymphoma tends to occur in older patients, with a mean age of 62 years, while secondary lymphoma occurs in relatively young people (10). As shown in Table 1, the mean age of patients with secondary PL was 59.8 ± 18.9, which was younger than patients with primary PL (59.8 ± 18.9 vs. 66.2 ± 8.8). Moreover, there are three conditions in which PL is determined to be primary (8): (1) presentation with symptoms attributable to prostatic enlargement; (2) involvement of the prostate gland predominantly, with or without involvement of adjacent tissue; and (3) absence of involvement of the liver, spleen, lymph nodes, or peripheral blood within 1 month of diagnosis of prostatic involvement. In the past, a needle biopsy was the standard method to identify whether extra-prostate tissue involvement was present, while it may lead to some complications (i.e., bleeding and infection). Recently, de Souza et al. pointed out that lymphoma may present as PSMA uptake after reviewing the data of 10 patients with different histological subtypes of lymphomas, and they subsequently found that the intensity of PSMA uptake was generally lower than FDG (11). Furthermore, several published case reports have also found that the PSMA expression of lymphoma was extremely low, or even had no expression (12–14). Thus, we deemed that the expression of PSMA on lymphoma was lower than adenocarcinoma, while the expression of FDG was higher. In our report, PSMA PET/CT was negative for lesions of the prostate gland and lymph nodes of the pelvic cavity and bilateral inguinal area, but FDG PET/CT showed higher glucose metabolism of lesions in the prostate gland with infiltration of the bladder wall and seminal vesicle. Moreover, multiple lymph nodes with increased glucose metabolism were observed, and the immunostaining further demonstrated that the lymphoma cells were negative for AR, PSA, and PSMA. Thus, our case did not satisfy these criteria based on the results of PSMA and FDG PET/CT and should be classified as secondary PL.

Prostate lymphoma is an uncommon entity in surgical practice and its diagnosis often poses considerable difficulty as it often mimics or is concurrent with carcinoma (15–17). Ultrasound, CT, and mp-MRI were the most commonly used imaging methods to detect PL and guide biopsy in the past. PSMA is a transmembrane protein that is considerably overexpressed in most prostate adenocarcinoma tumor cells (18). PSMA PET/CT was originally used to assess biochemical recurrence, and its clinical use has been recently extended to detection, restaging, and therapy response assessment of prostate adenocarcinoma. However, the diagnostic value of PSMA PET/CT in PL remains unclear. Miceli et al. reported a 38-year-old man with concomitant prostate adenocarcinoma and mediastinal lymphoma, and they found a high FDG-avidity and low PSMA uptake by mediastinal lymphadenopathies as opposed to the prostate adenocarcinoma (13). To our knowledge, this is the first report to clarify the appearance of PL on PSMA PET/CT and FDG PET/CT. Furthermore, we also found that PL was negative for PSMA, AR, and PSA, which also indicates that malignant neoplasms develop from lymph nodes or lymphatic tissues. Although PSMA had normal uptake in PL, PSMA PET/CT could non-invasively assist urologists in identifying the origin of lesions, revealing the characteristics of disease. Thus, we deduced that the combination of PSMA PET/CT and FDG PET/CT will benefit the discovery of the characteristics of PL. Because our report is a single case, further studies are needed to confirm this conclusion.

## Conclusion

In summary, secondary PL mostly occurs in relatively young patients, and its clinical symptoms are atypical. In addition to a DRE, PSMA PET/CT combined with FDG PET/CT may non-invasively help urologists identify the characteristics and origin of PL. Furthermore, systemic chemotherapy combined with local radiotherapy may be an optimal treatment regime to achieve more complete remission.

## Data Availability Statement

The original contributions presented in the study are included in the article/supplementary material, further inquiries can be directed to the corresponding author.

## Ethics Statement

Written informed consent was obtained from the patient for the publication of this case report (including all data and images).

## Author Contributions

FJ: data and material collection, literature search, and manuscript writing. JF: date collection, literature search, and manuscript writing. HL: literature search, pathological specimens review, and manuscript writing. XD: PET/CT data collection and manuscript writing. DH: project development and manuscript editing. KW: project development, operation performance, and manuscript editing. All authors contributed to the article and approved the submitted version.

## Conflict of Interest

The authors declare that the research was conducted in the absence of any commercial or financial relationships that could be construed as a potential conflict of interest.

## Publisher’s Note

All claims expressed in this article are solely those of the authors and do not necessarily represent those of their affiliated organizations, or those of the publisher, the editors and the reviewers. Any product that may be evaluated in this article, or claim that may be made by its manufacturer, is not guaranteed or endorsed by the publisher.
